# Bibliometric Analysis of Apoptotic Vesicles for Tissue Regeneration Research: Trends and Insights From 1991 to 2023

**DOI:** 10.1155/sci/3190427

**Published:** 2025-12-17

**Authors:** Guangzhao Tian, Zhen Yang, Haobin Deng, Xiang Sui, Shuyun Liu, Quanyi Guo

**Affiliations:** ^1^ Department of Orthopaedic Surgery, The Second Affiliated Hospital of Chongqing Medical University, No. 74 Linjiang Road, Yuzhong District, Chongqing, 400010, China, cqmu.edu.cn; ^2^ Institute of Orthopedics, Senior Department of Orthopedics, The Fourth Medical Center of PLA General Hospital, National Clinical Research Center for Orthopedics, Sports Medicine and Rehabilitation, 51 Fucheng Road, Haidian District, Beijing, 100142, China, 301hospital.com.cn; ^3^ Arthritis Clinical and Research Center, Peking University People’s Hospital, Beijing, China, pku.edu.cn; ^4^ Department of Oncology, Liuzhou People’s Hospital Affiliated to Guangxi Medical University, Liuzhou, China

**Keywords:** apoptotic vesicle, bibliometric, research trends, tissue regeneration, visualization

## Abstract

Apoptotic vesicles (ApoVs) have garnered considerable attention within the realm of tissue regeneration research, necessitating a comprehensive bibliometric analysis to delineate current international trends and to map out historical and contemporary developments in this domain.

**Methods:**

This study conducted a bibliometric analysis leveraging data sourced from the SCI‐Expanded Web of Science (WOS) database. The analysis encompassed publications from October 1, 1991, to December 31, 2023. A total of 1209 articles focusing on ApoVs for tissue regeneration were scrutinized, considering attributes such as publication year, journal, author, institution, country/region, references, and keywords. Coauthorship, cocitation, co‐occurrence analyses, network visualizations were generated using VOSviewer and CiteSpace.

**Results:**

The analysis indicated a steady annual rise in global publications pertaining to ApoVs for tissue regeneration. The United States emerged as the foremost contributor, with the highest citation count and H‐index. Furthermore, University of Tehran Medical Sciences was pinpointed as the most prolific institution. The journal *International Journal of Molecular Sciences* issued the largest account of articles on this topic. Notable subtopics such as “regenerative medicine,” “delivery,” and “mesenchymal stem cells” are poised to become significant research focal points in the near future.

**Conclusions:**

Over the past 30 years, research on ApoVs for tissue regeneration has witnessed substantial growth, mirroring increasing collaboration across various countries and institutions. This study illuminates trends, collaboration patterns, research hotspots, and future trajectories in the field, providing valuable insights for researchers and practitioners.

## 1. Introduction

Apoptosis is a regulated and systematic form of cell death. This natural process, often referred to as cellular suicide, is essential for the normal development and homeostasis of multicellular organisms [1]. A key characteristic of apoptosis is the fragmentation of the cell into small pieces that can be recognized and engulfed by neighboring phagocytes, such as macrophages, preventing the leakage of cellular contents and potential tissue damage or immune responses [1–4]. Apoptosis is also a prevalent phenomenon in both physiological and pathological processes. Physiologically, it occurs during embryonic development, cell differentiation, and tissue regeneration. Pathologically, it is observed in conditions such as tumors, immune deficiencies, and organ atrophy [5]. Apoptotic cells experience multiple biological events, such as cell shrinkage, nuclear fragmentation, chromatin condensation [6]. Ultimately, these changes result in the formation of membrane‐enclosed structures known as apoptotic vesicles (ApoVs), which are generated through budding or vesiculation [7]. These vesicles can be categorized based on their size: the larger membrane‐wrapped structures known as apoptotic bodies (ApoBDs/ABs) have diameters ranging from 1000 to 5000 nm [8]. The smaller vesicles, which are known as apoptotic microvesicles (ApoMVs) or exosome‐like ApoEVs, measure between 50 and 1000 nm in diameter [9]. It is noteworthy that ApoVs are not simply cellular remnants or merely incidental products of apoptosis [10]. These vesicles could be internalized by recipient cells and allow intracellular communication by transferring functional components [11, 12]. Specifically, ApoVs originating from mesenchymal stem cells (MSCs) are filled with miRNAomes, metabolites, and protein profiles, and have been shown to possess functions similar to MSCs, such as immunomodulatory effects and the ability to promote tissue regeneration. Growing evidence indicates that transplanted MSCs undergo significant apoptosis, where the released ApoVs function as crucial therapeutic agents [13–16]. MSC‐ApoVs not only facilitate immunostimulatory effects on various immune cells [17, 18], but are also considered promising options for promoting tissue repair in multiple disease models, including skin wounds, osteoporosis, and myocardial infarction [18–21]. Additionally, compared to the straightforward engraftment of MSCs, the use of MSCs‐ApoVs as a therapeutic approach can avoid potential issues associated with cell implantation, such as immune rejection, diminished regenerative capacity, tumorigenic risk, and certain ethical concerns [22]. Thus, the application of MSCs‐ApoVs in auxiliary diagnostics, vaccine creation, immune modulation, inflammation management, drug transport, and tissue repair is being extensively investigated as a promising new field of research [23, 24].

In the scientific research landscape, publication plays a pivotal role in gauging the contributions of scientific endeavors. To that end, bibliometric analysis has emerged as a valuable tool to assess research trends qualitatively and quantitatively over time, utilizing information from bibliometric databases and characteristics [25]. Additionally, such analyses have proven instrumental in formulating policies and clinical practice guidelines [26]. It has proven effective in examining research trends related to organoids in the field of diabetes [27], regenerative medicine [28], breast cancer [29], immunotherapy for thyroid cancer, and cutaneous neurofibromas [30]. However, the scope and quality of research specifically focused on ApoVs for tissue regeneration have not been adequately documented. Hence, this research seeks to carry out an in‐depth bibliometric analysis to assess the current state and project future developments in the field of “ApoVs for tissue regeneration.” Through this analysis, we aim to contribute to a more comprehensive understanding of the research landscape and foster progress in the area of aApoVs for tissue regeneration.

## 2. Materials and Methods

### 2.1. Data Source

Publication data are sourced from the SCI‐Expanded Web of Science (WOS), acknowledged as the premier bibliometric database [31].

### 2.2. Search Strategy

Relevant publications were obtained from WOS, with the search cutoff being December 10, 2023. The search utilized the following terms: theme = ((apoptosis body) OR (apoptosis vesicle) OR (apoptotic body) OR (apoptotic vesicle)) AND ((tissue repair) OR (tissue regeneration)) AND publishing year = (October 1, 1991, to December 31, 2023) AND Language = (English) AND Document types = (Article and review). Additionally, specific country or region data was refined by indexing them in WOS.

### 2.3. Data Collection

The criteria for including publications were defined in the following manner: manuscripts were required to focus on the topic of ApoVs for tissue regeneration; eligible document types encompassed articles and reviews; and publications were mandated to be in English. Conversely, the exclusion criteria encompassed themes not related to ApoVs for tissue regeneration, as well as types of documents including briefings, news pieces, and abstracts from meetings. The records of the selected publications, including the year of publication, article title, names of authors, institutional affiliations, countries or regions, abstracts, keywords, and names of journals, were retrieved from the SCI‐Expanded database. These records were stored as. txt files and later imported into Excel 2019 for organization. Data analysis was then performed using GraphPad Prism 9.0. Any encountered issues were resolved through consultation with domain specialists.

### 2.4. Bibliometric Analysis

The inherent features of the WOS defined the key characteristics of suitable publications. Furthermore, GraphPad Prism version 9.0 was utilized to visualize the yearly total of publications sourced from SCI‐Expanded. The relative research interest (RRI) was calculated as the ratio of publications in a specific field to the total publications in a given year. The world map was created by R software, with integration of Python, NumPy, SciPy, and matplotlib libraries. The publication time curve was developed using established methods [32]. GraphPad Prism 9.0 was used to examine publication statistics from the top 25 countries/regions, including total citations, average citation counts, and H‐index values. The H‐index, indicating that a researcher has published H papers each cited at least H times, served as a metric to evaluate the influence of scientific research, encompassing both the number of publications and the citation frequency [33]. Furthermore, Origin 2021 eased the analysis of high‐contributing journals, institutions, funding sources, and authors in the global landscape of publications pertaining to ApoVs for tissue regeneration.

### 2.5. Visualized Analysis

VOS viewer, a software tool developed by Leiden University, is a robust platform designed for mapping and visualizing citation networks. We utilized VOS viewer to generate visualizations of diverse networks, including those related to scholarly journals, research authors, geographical regions, and specific articles. These networks were built using bibliographic coupling, cocitation, coauthorship links, keyword co‐occurrences, as well as keyword co‐occurrences. Furthermore, we used Cite Space (Version 6.1 R2) to identify journals, authors, references, and keywords with burst citation activity, facilitating the anticipation of potential future hotspots and research frontiers.

## 3. Result

### 3.1. Overall Performance of Global Scholarship

**Figure 1:**
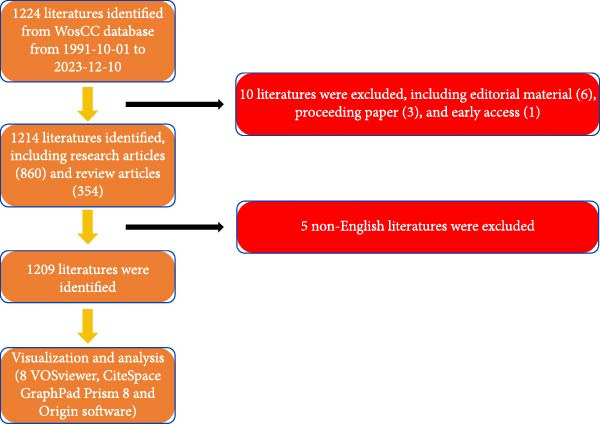
Flowchart depicting the literatures selection process.

In total, 1224 publications spanning from 1991 to 2023 were retrieved. After excluding editorial materials (6), proceeding papers (3), and early access articles (1), 1214 publications remained. Subsequently, 5 non‐English articles were filtered out, leaving a final count of 1209 original articles (Figure 1). Analysis depicted in Figure 2A illustrates a consistent upward trend in global literature over these years, with the total count of studies increased steadily from two in 1991 to 128 in 2023. Significantly, 2021 witnessed the highest publication output within the past decade, totaling 136 studies (Figure 2A). Moreover, there has been a marked increase in RRIin this field over recent years (Figure 2A). RRI values in this study range from 0 to 1, with 0 indicating no research attention, and 1 representing the highest level of focus. Specifically, a notable increase was observed from 2018 to 2021.

Figure 2Global trends and countries/regions contributing to the research field about apoptotic vesicles for tissue regeneration from 1991 to 2023. (A) The yearly publication count and RRI of studies related to apoptotic vesicles for tissue regeneration between 1991 and 2023. The green bars mean the number of publications each year, and the purple curve meas the RRI. (B) A world map illustrating the geographic distribution of research on apoptotic vesicles for tissue regeneration during the same period. The total number (C) and annual number (D) of publications in the top 10 most productive countries from 1991 to 2023.

**Figure:**
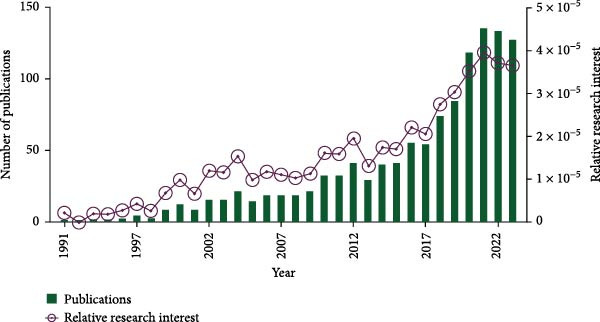
(A)

**Figure:**
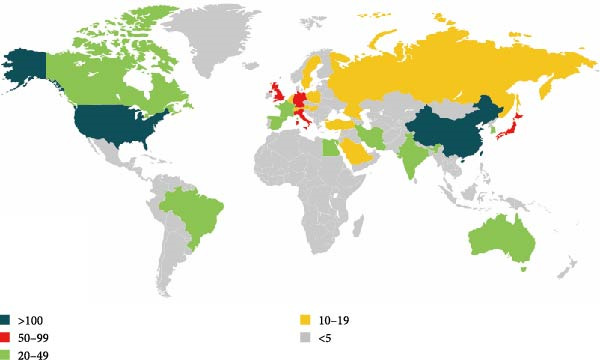
(B)

**Figure:**
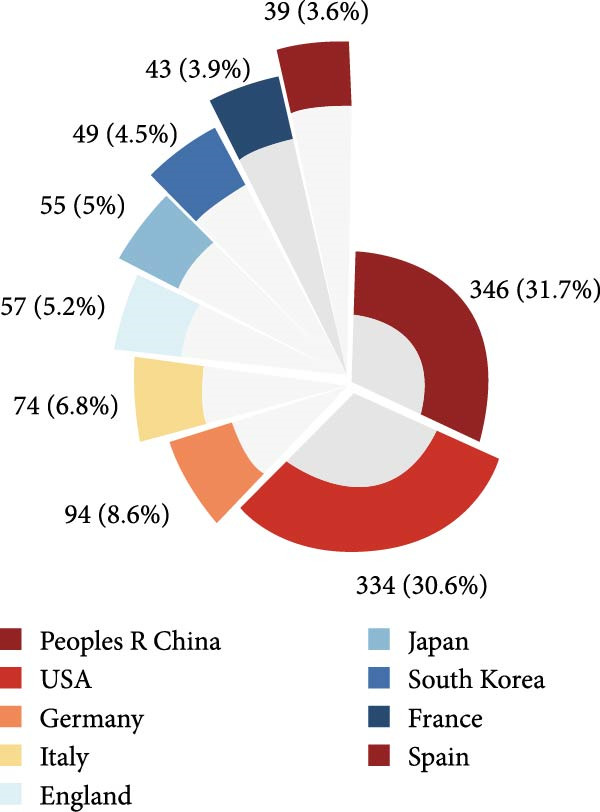
(C)

**Figure:**
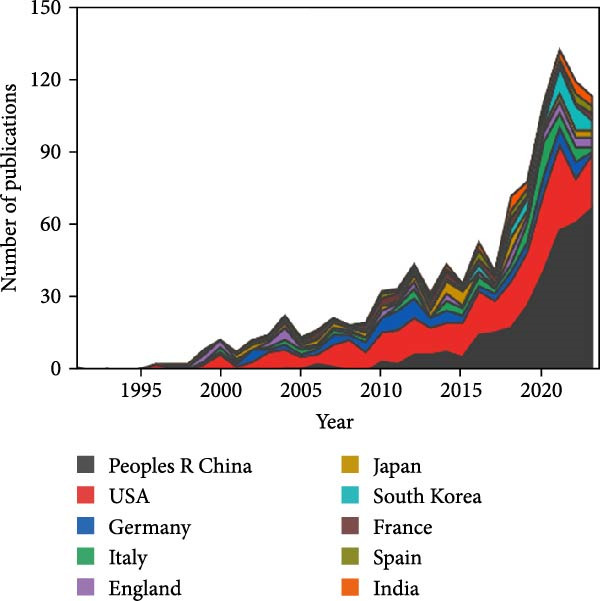
(D)

According to VOS viewer, contributions to the literature have been made by 69 countries/regions. As delineated in Figure 2B–D, China has become the primary contributor of papers (346), closely followed by the USA (334), Germany (94), Italy (74), and England (57). Figure 2C highlights that China and the USA dominate the publication landscape among the top 10 nations/areas, accounting for 31.7% and 30.6% of the total publications, respectively. Notably, over the last 5 years, there has been a significant rise in the annual publication rates across all countries (Figure 2D), indicative of escalating research interest and rapid advancements in the study of ApoVs for tissue regeneration.

### 3.2. Country Evaluation

In the chart shown in Figure 3A, the United States leads in total citation counts, with its papers garnering 23,199 citations. China secured the second position with 8146 citations, trailed by Germany (7067), Italy (3795), and England (3033). Notably, Germany led in average citation frequency, averaging 75.5 citations per publication. The USA followed closely with an average citation frequency of 69.5, succeeded by France (55.3), Italy (51.3), and Japan (38.1) (Figure 3B). Moreover, The USA (72) demonstrated leadership in H‐index publications within this field (Figure 3C), with China (45), Germany (39), England (32), and Italy (29) following suit.

Figure 3(A) Top 10 countries/regions with the highest total citations in research related to apoptotic vesicles for tissue regeneration from 1991 to 2023. (B) Top 10 countries/regions based on the average citations per publication in apoptotic vesicles for tissue regeneration research from 1991 to 2023. (C) Top 10 countries/regions ranked by the H‐index of publications on apoptotic vesicles for tissue regeneration from 1991 to 2023.

**Figure:**
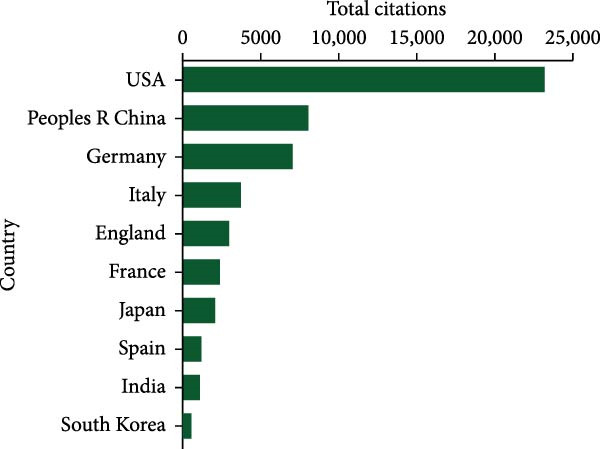
(A)

**Figure:**
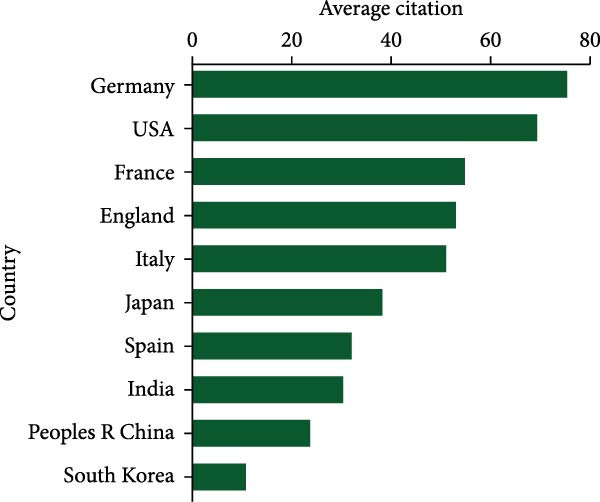
(B)

**Figure:**
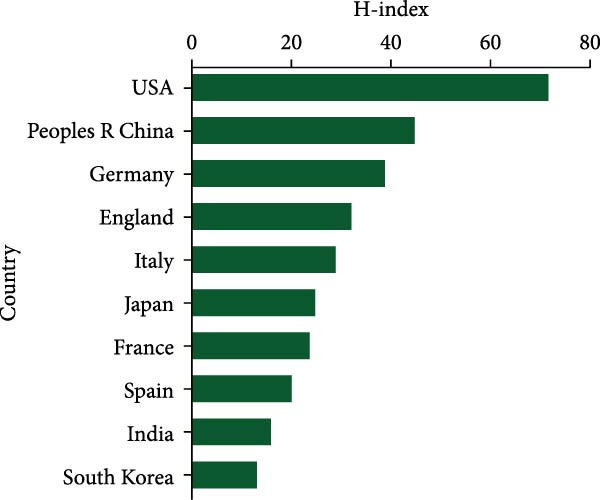
(C)

### 3.3. Analysis of International and Institutional Collaborations

Concerning the the global collaboration network and international authorship partnerships, Figure 4A,B illustrates the collaborative efforts among 54 countries. It is clear that the USA, China, and Germany engage in the most frequent international collaborations. Notably, Australia appears as a significant international partner for the USA. For institutional contributions, Table 1 ranks the top 10 institutions. As depicted in Figure 4C, Shahid Beheshti Univ Med Sci emerges as the key contributor to apoptotic vesicle research for tissue regeneration, as identified through bibliographic coupling analysis. Furthermore, Figure 4D visualizes the collaboration network among participating institutions. University of Tehran Medical Sciences emerges as the leading international collaborator, closely followed by Sechenov First Moscow State Medical University and Cairo University.

Figure 4Mapping of countries/regions/institutions involved in research on apoptotic vesicles for tissue regeneration from 1991 to 2023. (A) Country/regional collaboration analysis generated using Vosviewer. (B) Institutional collaboration analysis generated using Vosviewer. (C) Authorship‐country collaboration analysis conducted via Vosviewer. (D) Authorship‐institution collaboration analysis conducted via Vosviewer. The nodes represent countries/regions or institutions, and the lines connecting them illustrate collaborative relationships. The size of the nodes corresponds to the number of publications, while the thickness of the lines indicates the strength of collaboration; thicker lines represent closer collaborative relationships.

**Figure:**
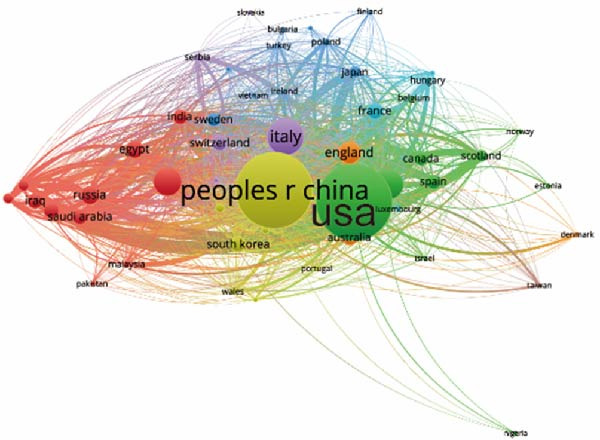
(A)

**Figure:**
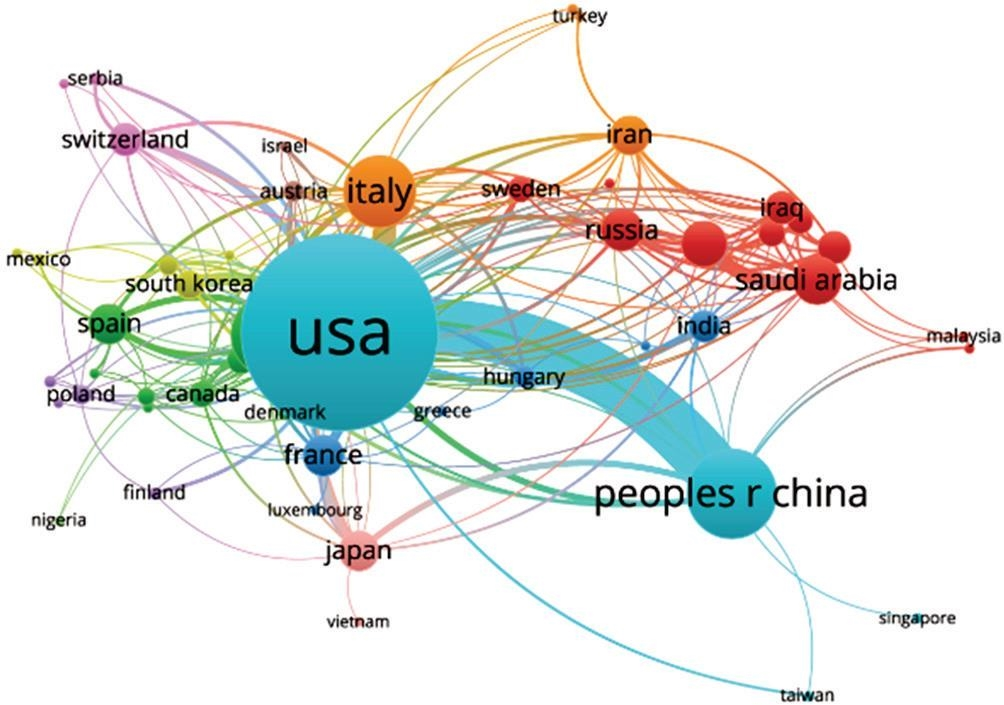
(B)

**Figure:**
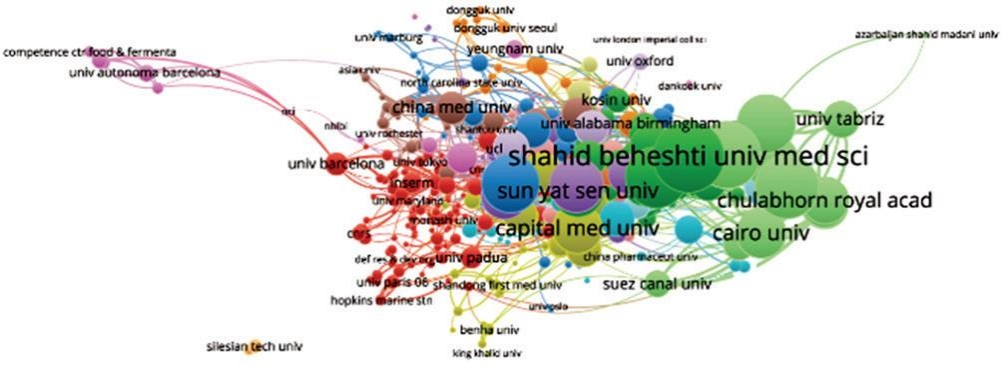
(C)

**Figure:**
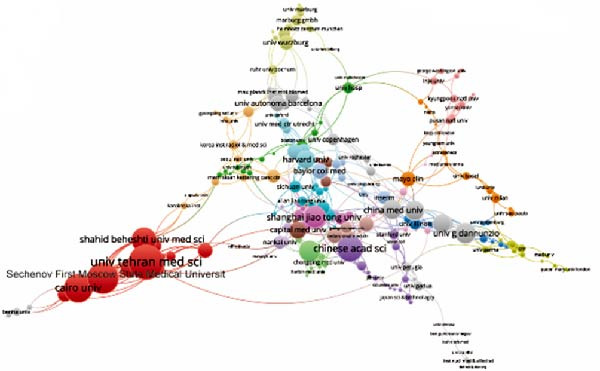
(D)

**Table 1:** The top 10 institutions published literature related to apoptotic vesicles for tissue regeneration from 1991 to 2023.

Rank	Institution	Article counts	Percentage	Country
1	Egyptian Knowledge Bank Ekb	32	2.653	Egypt
2	University of California System	28	2.322	USA
3	Shanghai Jiao Tong University	24	1.99	China
4	Institute National De La Sante Et De La Recherche Medical Inserm	22	1.824	France
5	University of London	19	1.575	England
6	University of Texas System	19	1.575	USA
7	Sichuan University	18	1.493	China
8	Harvard University	17	1.41	USA
9	Sun Yat Sen University	16	1.327	China
10	Chinese Academy of Sciences	15	1.244	China

### 3.4. Author Evaluation

In this study, a cohort of 405 authors within the field, each with a publication count exceeding two, underwent analysis using VOS viewer and Cite Space. The top 10 most prolific contributors are delineated in Table 2. Bibliographic coupling, a method using the shared citation of a third work, was employed to identify key contributors. As depicted in Figure 5A, Shiyu Liu appears with the highest aggregate link strength of 8571. Coauthorship relations, illuminating the collaborative dynamics among researchers, spotlight the top three authors with the greatest combined link strength: Camussi Giovanni, Shiyu Liu, and Hui Qian (Figure 5B). Delving into author cocitation via VOS viewer, those with a citation count exceeding 20 were scrutinized. As illustrated in Figure 5C, authors are depicted alongside their total link strength. The leading trio in this regard includes Thery C, Lai RC, and Bruno S. Figure 5D delineates a discernible temporal evolution of cited authors spanning from 2003 to 2023, showing a transition from Fadok Va to Barile L, and later to Zhang W during this timeframe.

Figure 5Network visualization of author collaboration analysis concerning apoptotic vesicles for tissue regeneration from 1991 to 2023. (A) Bibliographic coupling analysis of authors conducted using Vosviewer. (B) Network visualization diagram of authorship‐author analysis generated based on Vosviewer. (C) Network visualization diagram of cocited‐author analysis generated based on Vosviewer. (D) Top 25 cited authors exhibiting the strongest citation bursts in publications related to apoptotic vesicles for tissue regeneration. Author collaboration is indicated by nodes, with collaboration relationships represented by lines connecting the nodes. The area of the nodes corresponds to the number of collaborations; larger nodes indicate more extensive collaborations.

**Figure:**
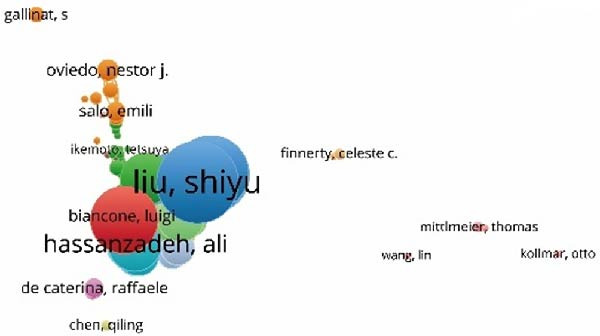
(A)

**Figure:**
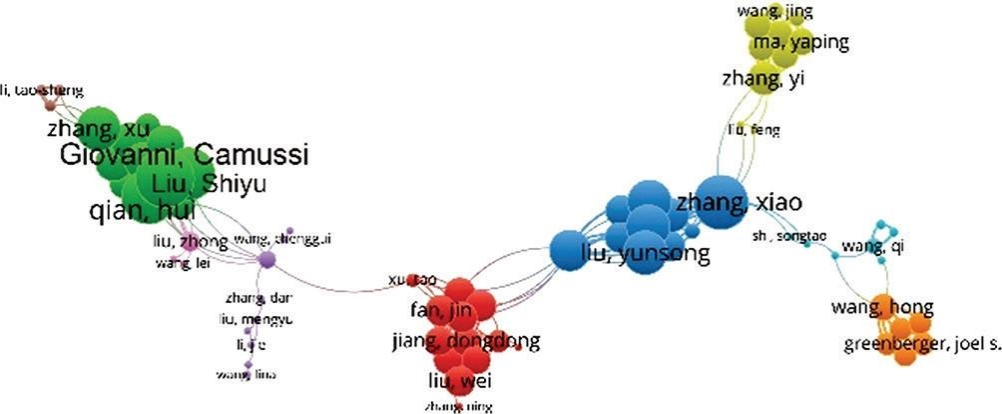
(B)

**Figure:**
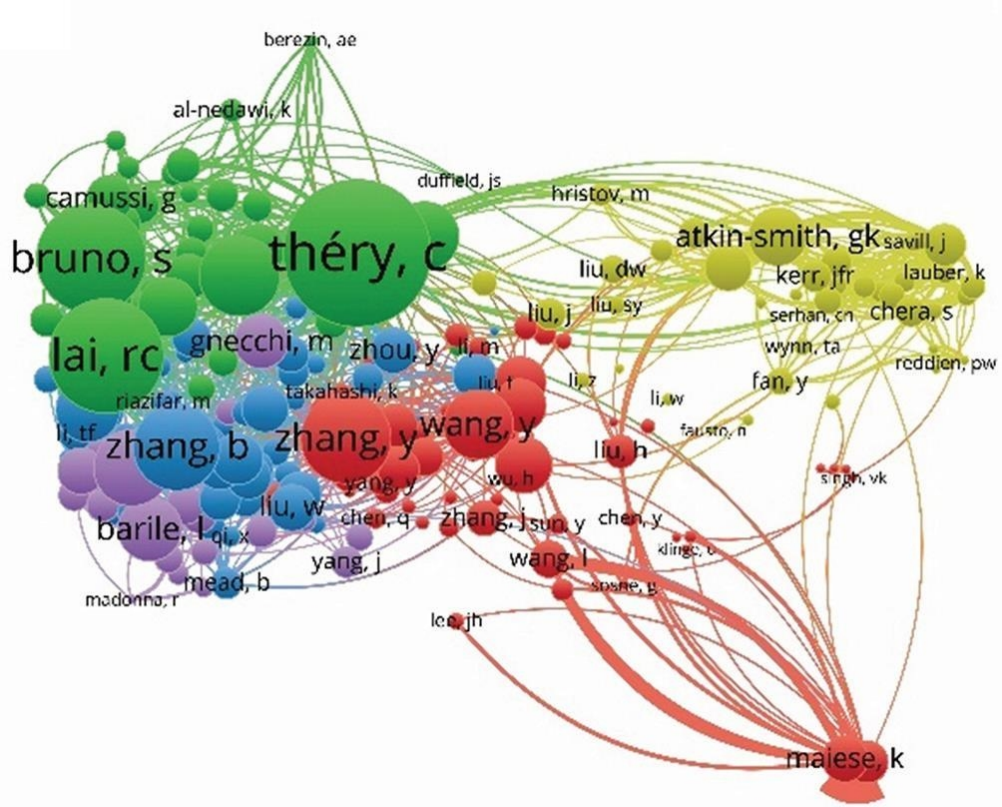
(C)

**Figure:**
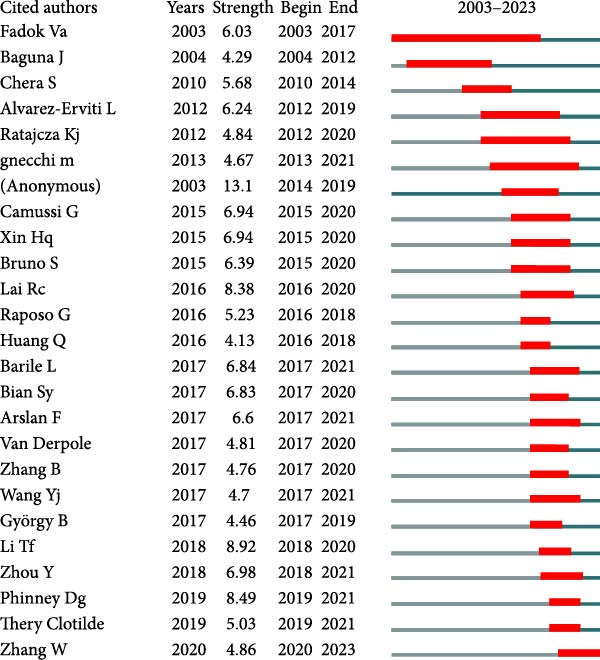
(D)

**Table 2:** The top 10 authors with the most publications on apoptotic vesicles for tissue regeneration from 1991 to 2023.

Rank	Highly published authors	Article counts	Percentage
1	Wang, Y	13	1.078
2	Wang, L	12	0.995
3	Li, Y	11	0.912
4	Camussi, G	10	0.829
5	Li, J	10	0.829
6	Wang, H	10	0.829
7	Zhang, Y	10	0.829
8	Liu, Y	9	0.746
9	Zhang, L	9	0.746
10	Gregory, CD	8	0.663

### 3.5. Research Areas and Journals Evaluation

Table 3 presents a comprehensive overview of the research domains, highlighting predominant areas such as cell biology, biochemistry molecular biology, and research experimental medicine. These delineate the current focal points and potential trajectories within the field.

**Table 3:** The top 10 well‐represented research areas.

Rank	Research areas	Records	Percentage
1	Cell Biology	291	24.129
2	Biochemistry Molecular Biology	166	13.765
3	Research Experimental Medicine	140	11.609
4	Science Technology Other Topics	106	8.789
5	Pharmacology Pharmacy	104	8.624
6	Materials Science	97	8.043
7	Engineering	77	6.385
8	Oncology	72	5.97
9	Chemistry	68	5.638
10	Biotechnology Applied Microbiology	63	5.224

In Table 4, we delineate the top 10 prolific journals pertinent to our study. Notably, the *Int J Mol Sci*leads with 32 publications, followed by *Stem Cell Res Ther* with 29 publications, and *Front Cell Dev Biol* with 20 publications. Employing bibliographic coupling, we examined the interdocument similarity relationships, finding 204 journals contributing to the total link strength (Figure 6A). Among these, the top 2 journals by total link strength were the *Int J Mol Sci* and *Front Cell Dev Biol*.

Figure 6Mapping of journals in studies on apoptotic vesicles for tissue regeneration from 1991 to 2023. (A) Bibliographic analysis of journals conducted using Vosviewer. (B) Network map of journals that were cocited, generated based on Vosviewer. (C) Top 25 cited journals exhibiting the strongest citation bursts in publications on apoptotic vesicles for tissue regeneration.

**Figure:**
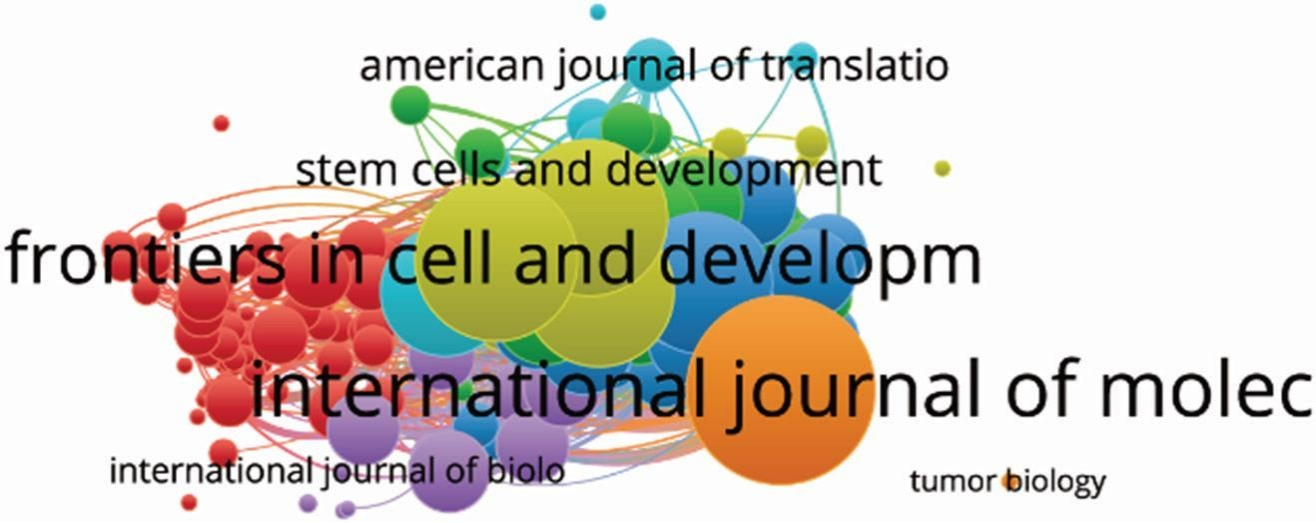
(A)

**Figure:**
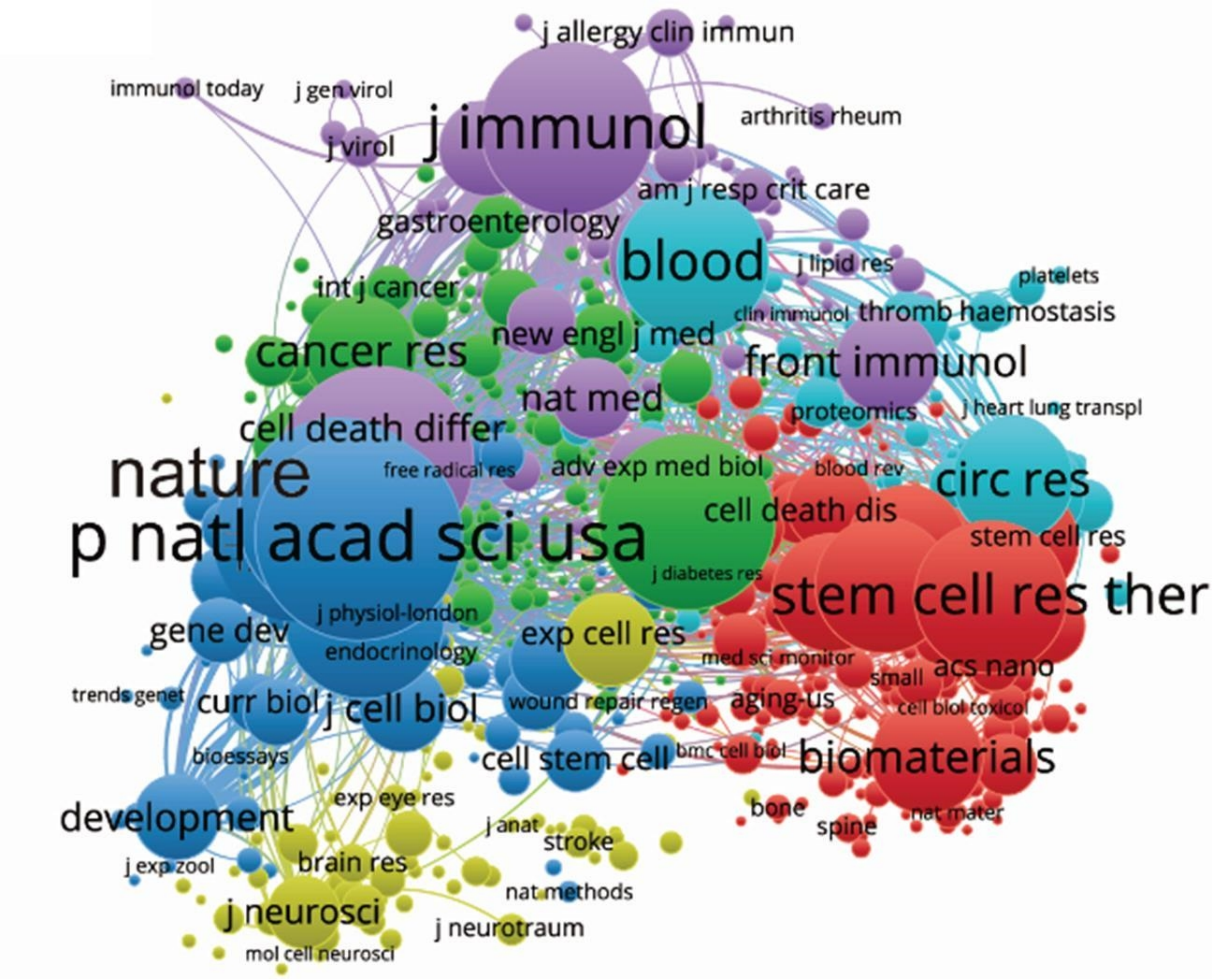
(B)

**Figure:**
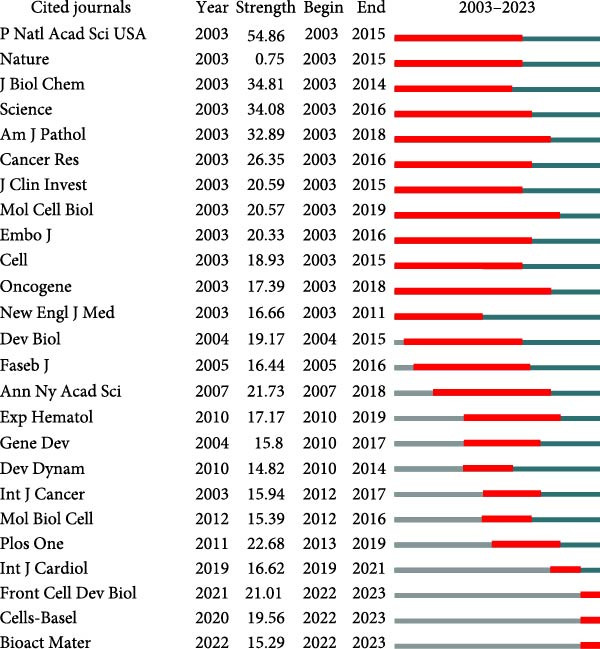
(C)

**Table 4:** The top 10 most productive journals related to apoptotic vesicles for tissue regeneration from 1991 to 2023.

Rank	Journal	Article counts	Percentage	Impact factor (IF, 2022)
1	International Journal of Molecular Sciences	32	2.653	5.6
2	Stem Cell Research and Therapy	29	2.405	7.5
3	Frontiers in Cell and Developmental Biology	20	1.658	5.5
4	Cells	17	1.41	5.5
5	Plos One	17	1.41	3.7
6	Biomaterials	15	1.244	14
7	Frontiers in Bioengineering and Biotechnology	15	1.244	5.7
8	Frontiers in Immunology	14	1.161	7.3
7	Scientific Reports	13	1.078	4.6
10	Acta Biomaterialia	11	0.912	9.7

Furthermore, employing VOS viewer for cocitation analysis, we identified journals with a minimum citation threshold of 20. As illustrated in Figure 6B, the top 2 journals by total link strength were *PNAS* and *Nature*. The visualization of journals exhibiting intense citation bursts over time (Figure 6C) serves as a valuable indicator of pivotal research dissemination hubs, potentially signifying shifts in academic influence and trends.

### 3.6. References Evaluation

Table 5 displays the top 5 highly cited research articles pertaining to ApoVs in tissue regeneration between 1991 and 2023. Notably, “Delivery of MicroRNA‐126 by Apoptotic Bodies Induces CXCL12‐Dependent Vascular Protection” accumulated 1059 citations, marking a significant impact. Furthermore, Table 6 presents the top 5 review articles in the same domain, with “Mast Cells” emerging as the most referenced review article. To find seminal works, cocited references underwent analysis using VOS viewer (Figure 7A). References having at least 10 citations were considered, revealing the top 3 with the highest total link strength: Thery C (2018) in J Extracell vesicles, Valadi H (2007) in Nat Cell Biol, and Lai RC (2010) in *Stem Cell Res*.

Figure 7Mapping of references in studies on apoptotic vesicles for tissue regeneration from 1991 to 2023. (A) A network map of reference analysis generated using Vosviewer, illustrating the connections and influence of key references in the field. (B) The top 25 references identified as having the strongest citation bursts, highlighting their significance and impact within the publication landscape of apoptotic vesicles for tissue regeneration.

**Figure:**
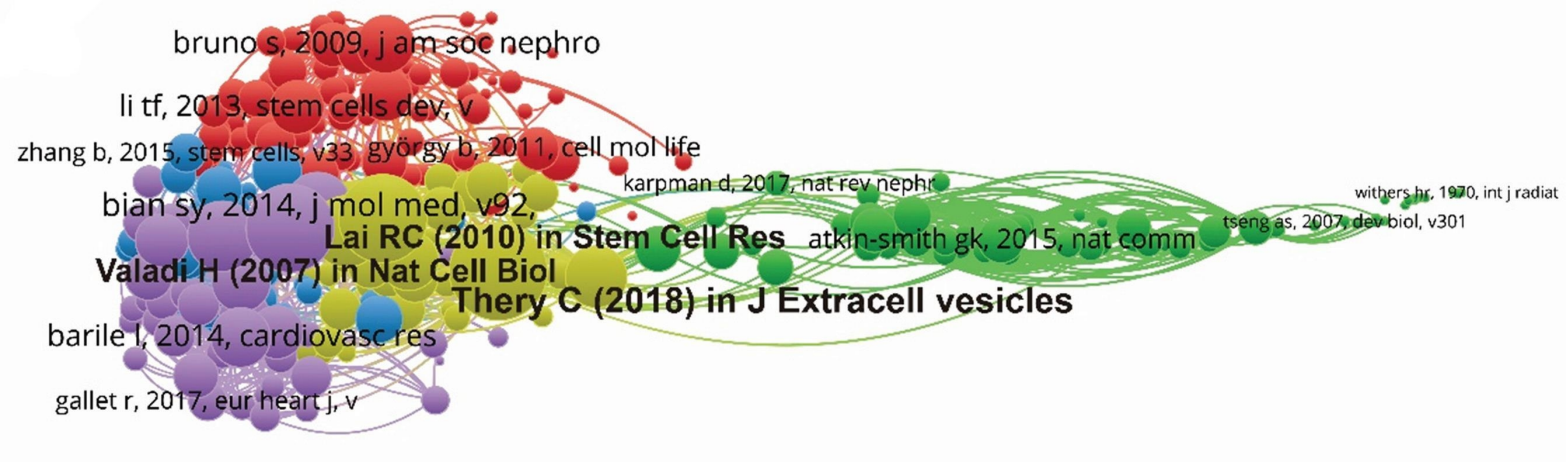
(A)

**Figure:**
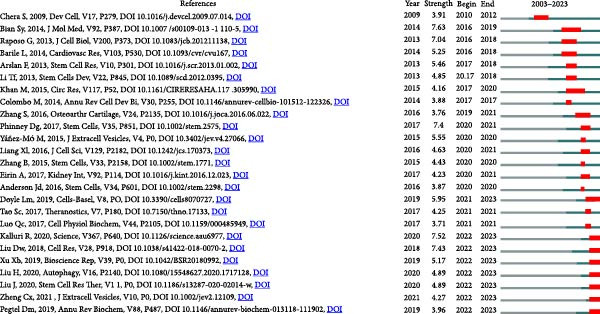
(B)

**Table 5:** The top 5 research articles with the most citations in the field of apoptotic vesicles for tissue regeneration from 1991 to 2023.

Rank	Title	First author	Journal	IF	Publication year	Total citations
1	Delivery of MicroRNA‐126 by Apoptotic Bodies Induces CXCL12‐Dependent Vascular Protection	Zernecke, A	Science Signaling	7.3	2009	1059
2	Kidney Injury Molecule‐1 is a Phosphatidylserine Receptor that Confers a Phagocytic Phenotype on Epithelial Cells	Ichimura, T	Journal of Clinical Investigation	15.9	2008	532
3	Extracellular Vesicles Derived From Human Bone Marrow MesenchymalSstem Cells Promote Angiogenesis in a Rat Myocardial Infarction Model	Bian, SY	Journal of Molecular Medicine‐JMM	4.7	2014	505
4	Recombinant Human Erythropoietin Protects the Myocardium From Ischemia‐Reperfusion Injury and Promotes Peneficial Remodeling	Calvillo, L	Proceedings of the National Academy of Sciences of the United States of America	11.1	2003	477
5	The Role of Complement in Inflammatory Diseases From Behind the ScenesIinto the Spotlight	Markiewski, MM	American Journal of Pathology	6	2007	467

**Table 6:** The top 5 review articles with the most citations in the field of apoptotic vesicles for tissue regeneration from 1991 to 2023.

Rank	Title	First author	Journal	IF	Publication year	Total citation
1	Mast Cells	Metcalfe, DD	Physiological Reviews	33.6	1997	1697
2	Bone Morphogenetic Proteins: Multifunctional Regulators of Vertebrate Development	Hogan, BLM	Genes and Development	10.5	1996	1664
3	Programmed Cell Death in Animal Development and Disease	Fuchs, Y	Cell	64.5	2011	1247
4	Wound Repair and Regeneration	Reinke, JM	European Surgical Research	1.6	2012	1075
5	Toxic Mechanisms of Five Heavy Metals: Mercury, Lead, Chromium, Cadmium, and Arsenic	Balali–Mood	Frontiers in Pharmacology	5.6	2021	555

Moreover, citation burst analysis serves as a crucial metric indicating the surge in interest among researchers within a specific domain over time. In our investigation, Cite Space identified the top 25 references with the most substantial citation bursts, depicted in Figure 7B, illustrating the burst duration. Notably, the article authored by Bian SY in 2014 and published in J Mol Med maintained the strongest citation burst, with a size of 7.63, spanning from 2016 to 2019.

### 3.7. Keyword and Hotspot Evaluation

The aim of co‐occurrence analysis is to explore prevalent research trends and domains, serving as a crucial tool for tracking advancements in scientific inquiry. Employing VOS viewer, we examined keywords, defined as terms recurring over 20 times in titles/abstracts across all papers. Figure 8A presents the depiction of 484 identified keywords, with central keywords such as expression, repair, inflammation, and stem cells showing greater prominence, indicative of their higher relevance. Thus, there remains a call for further robust investigations into ApoVs for tissue regeneration within these thematic directions. Furthermore, employing color‐coded representation in Figure 8B, keywords were categorized based on their average occurrence across published papers. Purple hues denote keywords prevalent in earlier literature, while yellow hues signify more recent appearances. Notably, MSCs appear prominently in recent keyword analyses.

Figure 8Mapping of keywords in studies on apoptotic vesicles for tissue regeneration from 1991 to 2023. (A) Network visualization of keywords generated using Vosviewer, where the size of the points represents the frequency of keyword occurrence. (B) Distribution of keywords based on the mean frequency of their appearance; keywords shown in yellow emerged more recently compared to those in blue. (C) Keyword clustering visualization, illustrating the thematic grouping of keywords from 1991 to 2023. (D) The top 25 keywords exhibiting the strongest citation bursts, indicating their sudden increase in prominence and impact within the field.

**Figure:**
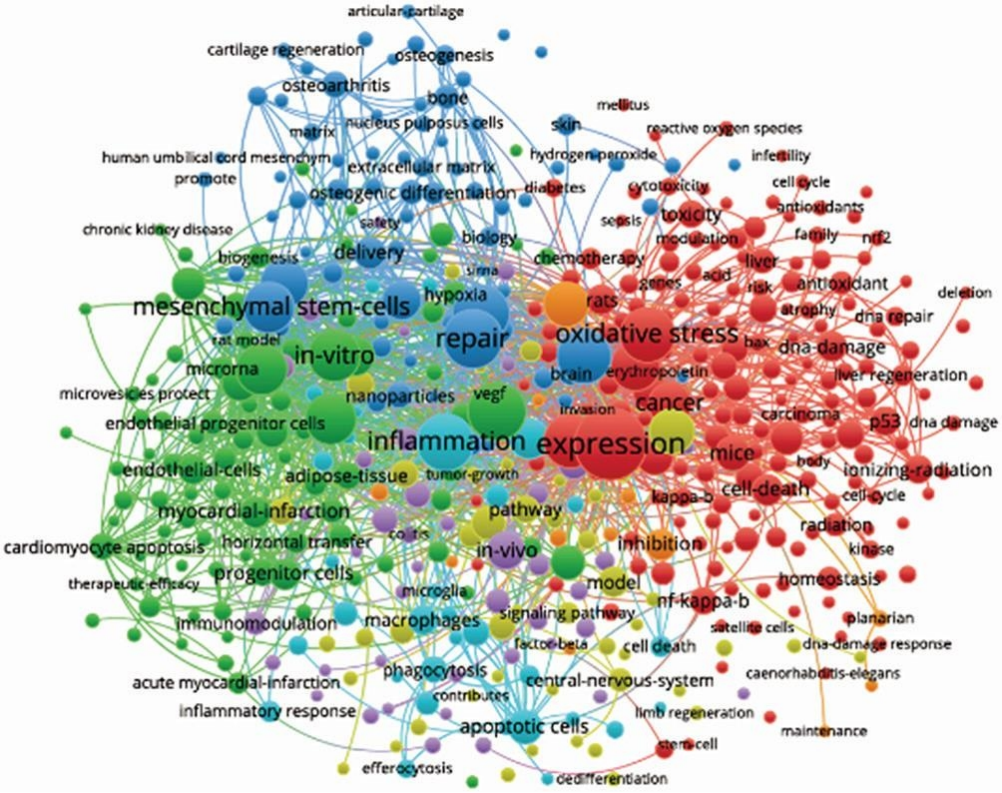
(A)

**Figure:**
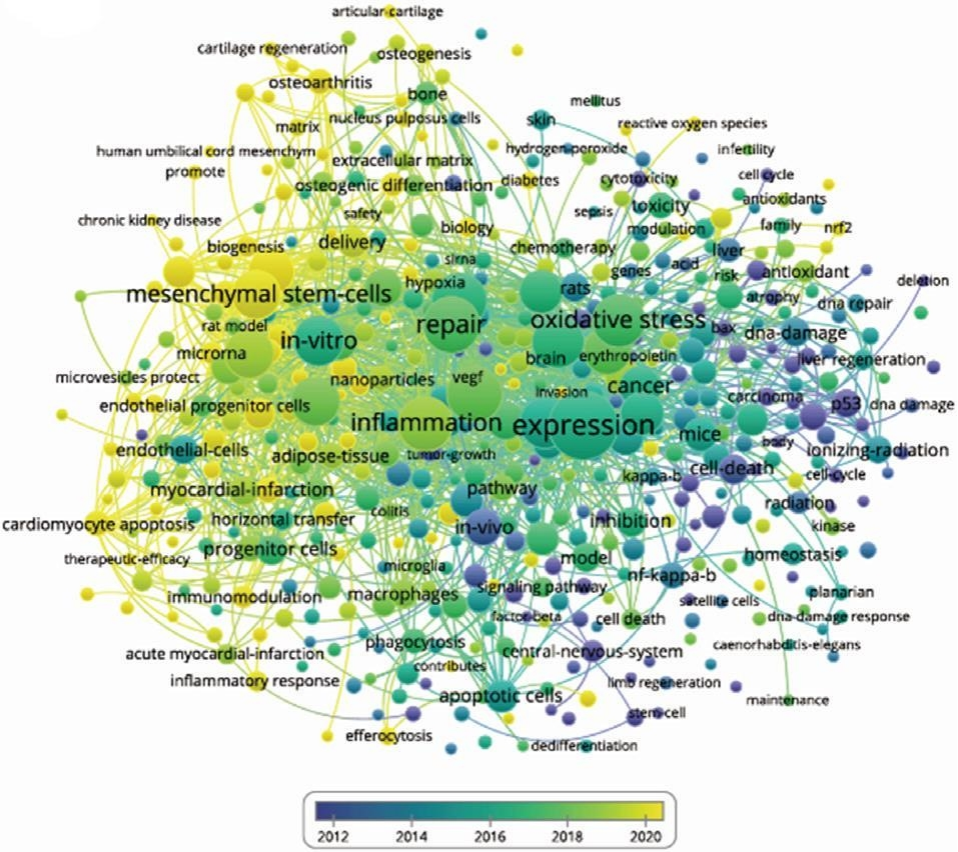
(B)

**Figure:**
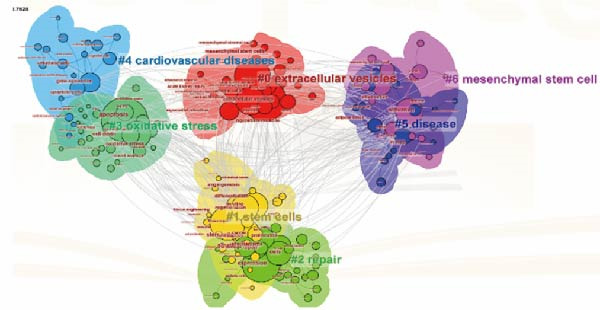
(C)

**Figure:**
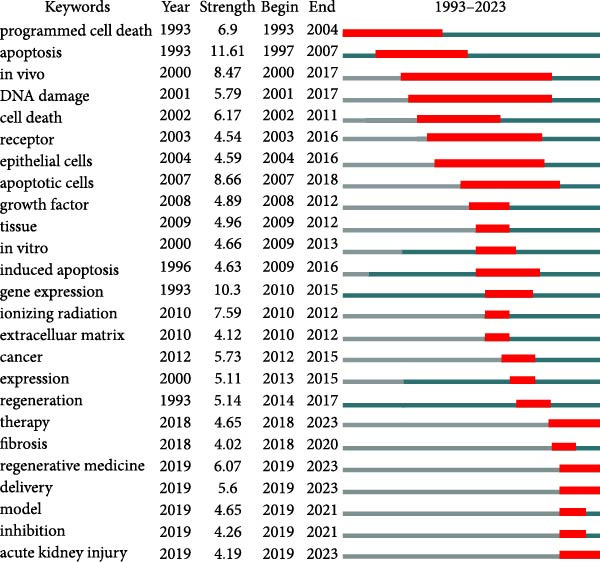
(D)

By clustering cocited documents, we gain insights into document interconnections, offering valuable perspectives on the document landscape. Thus, we conducted cocitation analysis focusing on ApoVs for tissue regeneration to unveil historical trajectories and scientific contexts. This analysis yielded seven primary clusters, each labeled with phrases extracted from abstracts (Figure 8C).

Keywords showing powerful bursts within a concise timeframe serve as sensitive indicators of evolving research emphases. Recent burst keywords signal potential short‐term research frontiers. Utilizing Cite Space, we generated a keyword burst map, illustrating burst strength and onset or cessation years (Figure 8D). Burst strength indicates intensity, while the burst year denotes shifts in research focus and duration. Among the most notable bursts, apoptosis (strength = 11.61) leads, followed by gene expression (10.3) and apoptotic cells (8.66). Moreover, keywords such as “therapy,” “regenerative medicine,” “delivery,” and “acute kidney injury” have experienced recent outbreak citations (2018–2023), suggesting their potential as future research hotspots.

## 4. Discussion

Bibliometric analysis coupled with visual examination offers insights into the prevailing trends within the research domain and facilitates prognostication [32]. The goal of this research is to assess ApoVs for tissue regeneration, focusing on authors, donor countries, journals, institutions, and research areas within the open access field. Over the course of the last three decades, the advancement of ApoVs as a therapeutic modality for tissue regeneration has emerged as an increasingly dynamic and promising area of investigation.

### 4.1. Trends in ApoVs for Tissue Regeneration Research

The consistent annual growth in publications (from 2 in 1991 to 128 in 2023) reflects the growing recognition of ApoVs as a pivotal element in tissue regeneration research. The notable surge in RRI between 2018 and 2021 (Figure 2A) aligns with significant advancements in understanding ApoVs functions, such as their ability to transfer miRNAs and proteins for intercellular communication [11, 12], and their proven therapeutic efficacy in models like skin wounds and myocardial infarction [19, 21]. This indicates that preclinical breakthroughs have been a key driver of increased academic attention.

### 4.2. Global Publication Quality and Standing

China leads in the number of publications (346), while the United States excels in quality metrics, including total citations (23,199) and H‐index (72). This difference may result from varying research focuses: U.S. studies tend to explore fundamental mechanisms (e.g., ApoVs biogenesis [9]), while Chinese research emphasizes translational applications (e.g., hyaluronic acid hydrogel‐mediated ApoV delivery for intrauterine adhesions [19]). Germany’s high average citations (75.5) highlight its impact in specialized areas.

The bibliographic coupling analysis establishes the USA as the primary country, while the Univ Tehran Med Sci stands out as the premier institution in this field. Coauthorship analysis underscores the collaboration between countries, institutions, and researchers, with the USA and prominent research institutes leading in ApoVs for tissue regeneration research.

The multidisciplinary nature of the field is evident, involving cell biology, biochemistry molecular biology, pharmacology, material science, engineering, and oncology. Prominent journals like *Int J Mol Sci*, *Stem Cell Res Ther*, *and Front Cell Dev Biol* play essential roles in disseminating research. However, articles are relatively scattered among various journals, suggesting the potential for new discoveries in specialized outlets.

Citation analysis emphasizes the impact of published literature. With the article “Mast Cells” standing out as the most cited, shedding light on research, which may be the beacon light for ApoVs for tissue regeneration research. Additionally, “Minimal Information for Studies of Extracellular Vesicles 2018 (MISEV2018): A Position Statement of the International Society for Extracellular Vesicles and Update of the MISEV2014 guidelines” emerges as a highly influential reference in the field [34].

### 4.3. The Research Emphasis and Future Directions of ApoVs in Tissue Regeneration

Keyword analysis can indirectly uncover the main research topics and features within the field. Using co‐occurrence analysis, we identified the emerging trends and popular topics. The co‐occurrence map (Figure 8A) highlights two distinct research directions. Although these findings are in line with existing knowledge, this study helps to further define potential areas for future exploration. As displayed clearly in the central part of the map, keywords include “expression,” “repair,” “oxidative stress,” “inflammation,” and “MSCs,” etc. have a greater weight indicating frequency. Colors were assigned to the overlay map by VOS viewer based on the average frequency of keyword appearances. This method is crucial for guiding research monitoring. In the overlay visualization presented in Figure 8B, the color indicates the year of publication. Combined with keyword clustering analysis (Figure 8C), We identified that the earliest research clusters focusing on ApoVs for tissue regeneration were “extracellular vesicles (EVs)” and “stem cells,” followed by “repair.” Furthermore, in recent years, studies focusing on “disease” and “MSC” have been on the rise and are likely to garner significant attention in the coming years.

Burst keywords highlight the evolving research focus, shifting from the apoptosis mechanism to therapeutic applications. The persistence of burst keywords indicates emerging trends and promising developments in apoptotic vesicle research for tissue regeneration (Figure 8D). Keyword bursts from 2018 to 2023, such as “regenerative medicine” and “delivery,” correspond to the practical advantages of ApoVs over exosomes, including easier isolation and higher secretion efficiency [7], making them suitable for large‐scale therapeutic development. The increasing focus on “MSC‐derived ApoVs” (Figure 8B) indicates a shift toward cell‐type‐specific vesicles, leveraging MSCs’ regenerative properties while avoiding cell transplantation risks like immune rejection [22].

Some clinical trials involving MSCs‐exosomesand MSCs‐microvesicles have been conducted, and these trials have shown promising potential for these bioactive substances in tissue regeneration and disease treatment. The preclinical significance of MSCs‐ApoVs in tissue regeneration, as discussed earlier, suggests their potential utility in regenerative therapies. Furthermore, ApoVs‐based regenerative therapy offers several advantages over exosome‐based approaches. Firstly, ApoVs are more accessible and easier to isolate from bodily fluids due to their high abundance and simple extraction process [7]. Secondly, ApoVs exhibit higher secretion efficiency compared to exosomes. While exosomes are formed by the inward budding of endosomal membranes, creating vesicles inside multivesicular bodies, the process occurs within the endosomal system [9]. Additionally, ApoVs may possess greater therapeutic potency. For instance, mature osteoclast‐derived ApoVs demonstrated superior osteogenic potency compared to exosomes, micro vesicles, and healthy cell counterparts [35]. In another study, the isolation of ApoVs required fewer huMSCs, and the implantation of huMSCs‐ApoVs resulted in superior endometrial regeneration compared to exosome‐based treatments [19]. Moreover, ApoVs transplantation might provide a safer option compared to exosomes, as they trigger fewer inflammatory reactions and graft rejections [36].

As previously indicated, the inherent phagocyte targeting property of ApoVs could potentially facilitate phagocyte‐specific drug delivery. These vesicles can transport biologically active cargo, including DNA, miRNA, and proteins, thereby facilitating intercellular communication upon transfer to recipient cells. ApoVs, derived from the cell membrane, exhibit a lipid bilayer structure similar to cell membranes and contain a higher concentration of surface‐integrated protein molecules compared to exosomes, which originate from multivesicular endosomes within the cytoplasm. Recently, researchers have adopted a bionic nanosphere construction method to harness the benefits of both ApoVs and artificial nanomaterials [37]. For instance, Geng et al. developed chimeric ApoVs by enhancing their surface with the native ApoVs membrane and incorporating a versatile mesoporous silica nanoparticle delivery platform to control inflammation [38]. Furthermore, the potential for genetically engineering cells to generate ApoVs with targeting or therapeutic functionalities is significant, coupled with the ability to chemically modify or load these ApoVs with cargo, presents promising opportunities for utilizing ApoVs as a versatile drug delivery platform. While these innovative engineering strategies have garnered considerable attention in the realms of EV‐based immunotherapy and antitumor therapy [39, 40], their exploration in the context of ApoVs‐based regenerative therapy remains relatively limited.

### 4.4. Strengths and Limitations

This study analyzed publications retrieved from the SCI‐Expanded database within (WOS) to ensure the reliability and objectivity of its findings. As the first bibliometric analysis specifically focusing on ApoVs in tissue regeneration, it clearly differentiates ApoVs from other EVs and elaborates on their unique mechanisms and application value.

By integrating network analysis via VOSviewer and burst detection through CiteSpace, the research uncovers hidden trends in the field—such as the shift in focus from “apoptosis mechanisms” to “therapeutic delivery”—and categorizes existing studies into seven thematic clusters. These insights not only identify underexplored areas, including research on ApoVs in neuroregeneration, but also highlight the bridging role of trends like “drug delivery” and “tissue engineering” between basic research and clinical practice. Ultimately, this work provides guidance for future efforts in scalable ApoVs production and the development of targeted delivery systems.

Despite these efforts, our study has several limitations. Publications from various databases like WOS, PubMed, Embase, and the Cochrane Library differ significantly, leading to potential omissions due to database bias. Furthermore, our reliance on the English language search strategy within the SCI‐Expanded database may have excluded non‐English literature, resulting in a potential language bias. Given the database’s ongoing updates, there might be slight discrepancies between our results and real‐world findings. Another limitation of this study is the absence of manual validation for key nodes, including country clusters and author impact, which are core components of our bibliometric analysis. Last, the lack of standardized parameter settings in VOSviewer may cause variations in cluster analysis results depending on the chosen settings.

## 5. Conclusion

The purpose of this study is to systematically map the global research landscape of ApoVs in tissue regeneration from 1991 to 2023 through bibliometric analysis, aiming to quantify publication trends, geographical contributions, collaboration patterns, identify key institutions, journals, and authors shaping the field, and uncover research hotspots and future directions to guide researchers and practitioners. The key conclusion is that research on ApoVs for tissue regeneration has experienced steady growth over the past 32 years, with accelerating interest since 2018 driven by preclinical successes and interdisciplinary collaboration; the United States leads in research quality (as indicated by citations and H‐index), while China dominates in publication quantity, with the United States, China, and Germany forming major collaborative hubs; the University of Tehran Medical Sciences and  ^∗^Int J Mol Sci ^∗^ are the most prolific institution and journal in this field, respectively; and future research is likely to focus on “regenerative medicine,""drug delivery,” and “MSC‐derived ApoVs,” reflecting a shift toward translational applications and engineered ApoVs systems.

## Ethics Statement

The authors have nothing to report.

## Consent

The authors have nothing to report.

## Conflicts of Interest

The authors declare no conflicts of interest.

## Author Contributions

Guangzhao Tian, Zhen Yang, Haobin Deng, Shuyun Liu, and Quanyi Guo were involved in the conception and design of the study. Guangzhao Tian and Zhen Yang collected the data and conducted the data analysis and quality assessment. Guangzhao Tian prepared the initial draft of the manuscript, while Zhen Yang and Xiang Sui authors reviewed and provided critical revisions for its intellectual content. All authors significantly contributed to the interpretation of the findings. All authors have approved the final version for submission. Guangzhao Tian and Zhen Yang contributed to this work equally.

## Funding

This study was funded by the Natural Science Foundation of Beijing Municipality (L234024).

## Data Availability

The data that support the findings of this study are available from the corresponding author upon reasonable request.
